# Safety and efficacy of tirzepatide in transplant recipients: a systematic review and meta-analysis

**DOI:** 10.3389/fphar.2026.1735987

**Published:** 2026-03-13

**Authors:** Alessio Provenzani, Brinn Mancuso, Riley Stitch, Fabio Tuzzolino, Maria Ausilia Giusti, Alessandro Mattina

**Affiliations:** 1 IRCCS ISMETT, Palermo, Italy; 2 UPMC Italy, Palermo, Italy; 3 Department of Pharmacy and Therapeutics, University of Pittsburgh School of Pharmacy, Pittsburgh, PA, United States

**Keywords:** adverse drug reactions, efficacy, safety, tirzepatide, transplant recipients

## Abstract

**Introduction:**

Tirzepatide has demonstrated cardiovascular and metabolic benefits in the general population; however, evidence in post-transplant patients is very limited. The aim of this systematic review and meta-analysis is to evaluate the safety and efficacy of tirzepatide in solid organ transplant recipients.

**Methods:**

We searched PubMed-MEDLINE, Embase, and Cochrane Library databases. All randomized controlled trials (RCTs) and observational studies were considered. Efficacy outcomes included improvements to glycemic outcomes demonstrated by reductions to hemoglobin A1c and changes to weight, measured by body mass index. Safety was assessed through patients who discontinued tirzepatide treatment due to adverse drug reactions.

**Results:**

No randomized controlled trials (RCTs) or other interventional clinical trials were identified in the available literature. Four non-randomized observational studies were found and included. Using the Weighted Median of the Difference of Medians statistical test, tirzepatide was associated with absolute reductions in hemoglobin A1c of −1.4% (95% CI: −1.7 to −0.4) and body mass index of −1.2 kg/m^2^ (95% CI: −5.9 to −1.1) in solid organ transplant recipients. Pooled proportions indicated a tirzepatide discontinuation rate of 3.1% (95% CI: 0.0–7.1) due to adverse drug reactions, suggesting the therapy was well tolerated in this population.

**Conclusion:**

Tirzepatide was associated with reductions in hemoglobin A1c and body mass index and was generally well tolerated in solid organ transplant recipients. These findings suggest a potential role for tirzepatide in the management of obesity and post-transplant diabetes mellitus, pending confirmation in larger prospective studies.

**Systematic Review Registration:**

https://www.crd.york.ac.uk/PROSPERO/view/CRD420251154851, identifier CRD420251154851.

## Highlights

- Solid organ transplant recipients are at an increased risk of post-transplant complications, such as obesity and post-transplant diabetes mellitus; however, pharmacologic treatments to manage these complications are limited by drug interactions and metabolic dose adjustments.
- In post-transplant patients across 4 retrospective chart reviews, tirzepatide was associated with clinically meaningful reductions to hemoglobin A1c and modest but consistent decreases to body mass index with limited discontinuations due to adverse events.
- Despite several limitations to our review, tirzepatide has demonstrated a promising role in post-transplant patients to manage complications and prevent the progression of metabolic disease through hemoglobin A1c and weight reductions while maintaining safety.

## 1 Introduction

According to the most recent data from the Global Observatory on Donation and Transplantation, a total of 172,409 solid organ transplants (SOT) were performed worldwide in 2023 (Global Observatory on Donation and Transplantation GODT, 2023). This number is increasing year by year, paralleled by improvements in patient survival and the rising age of transplant recipients. Such progress, largely driven by advances in surgical techniques and immunosuppressive (IS) therapies, has also expanded the pool of transplant recipients who can be considered a medically fragile population at high risk of developing non-communicable diseases (NCDs) (Sen et al., 2019). In particular, long-term IS regimens contribute substantially to cardio-renal-metabolic complications, including obesity and post-transplant diabetes mellitus (PTDM). The term PTDM was established at the 2013 International Consensus Meeting to describe diabetes associated with immunosuppression, regardless of the timing of onset (Sharif et al., 2014). Like type 2 diabetes mellitus (T2DM), PTDM is characterized by the dual pathogenic mechanisms of insulin resistance and β-cell dysfunction (Chastagner et al., 2024; Sharif and Cohney, 2016). However, in PTDM both pathways are directly affected by IS therapy, with calcineurin inhibitors (CNIs) and corticosteroids playing a predominant role, accelerating the onset and exacerbating disease severity (Sharif and Cohney, 2016).

PTDM occurs in 10%–40% of SOTRs (Liu et al., 2024), with risk factors including age, family history of diabetes, genetic predisposition, post-surgical hyperglycemia, donor and recipient characteristics, treatment of acute rejection, and post-transplant lifestyle changes such as weight gain and increased visceral adiposity (Sharif et al., 2014; Chastagner et al., 2024). Per the American Diabetes Association (ADA) guidelines, the oral glucose tolerance test (OGTT) is the preferred screening tool to confirm a diagnosis of PTDM (American Diabetes Association Professional Practice Committee. 9, 2025). The ADA guidelines also suggest metformin, glucagon-like peptide-1 receptor agonists (GLP1 RAs), sodium-glucose cotransporter-2 inhibitors (SGLT2i), dipeptidyl peptide-4 (DPP-4) inhibitors, and pioglitazone have demonstrated safety and efficacy in heart, kidney, and liver transplant recipients (American Diabetes Association Professional Practice Committee. 9, 2025). However, most available studies are constrained by limited cohorts, brief observation periods, and methodological bias related to retrospective or single-arm prospective designs. In addition, certain agents may not be ideal given the frequent fluctuations in renal and hepatic function observed in transplant recipients. Among the available options most widely used in T2DM, incretin-based therapies, such as GLP-1 RAs, are emerging as potentially preferred agents to manage PTDM and other metabolic post-transplant complications, as they provide cardiovascular, renal, and hepatic benefits as well as reductions in body weight (American Diabetes Association Professional Practice Committee. 9, 2025; Zheng et al., 2024).

Glucagon-like peptide-1 (GLP-1) is an incretin hormone secreted in response to elevated plasma glucose concentrations associated with food intake (Zheng et al., 2024; Andreasen et al., 2021). Its release stimulates insulin secretion while inhibiting glucagon secretion to lower plasma glucose levels (Zheng et al., 2024). GLP-1 also results in appetite suppression through delayed gastric emptying and induction of early satiety through interactions with receptors in the gastrointestinal tract and hypothalamus. Glucose-dependent insulinotropic polypeptide (GIP) is another hormone responsible for the incretin effect, believed to stimulate additional insulin secretion. GIP exerts also peripheral effects on bone and adipose tissue, where it acutely suppresses bone resorption and promotes lipid storage through anabolic actions in adipocytes, a paradoxical effect which, in combination with GLP-1R agonism, appears to reprogram signaling in a manner that reduces adiposity rather than promoting it (Nissen et al., 2014; Asmar et al., 2010). More recent preclinical evidence further suggests that central activation of GIPR-expressing neurons in the hypothalamus can decrease food intake and facilitate weight loss (Adriaenssens et al., 2019).

These mechanisms have led to the development of incretin-based therapies, such as GLP-1 receptor agonists (RAs) and GLP-1/GIP RAs, which are revolutionizing the management of diabetes mellitus (DM) and obesity. GLP-1 RAs mimic endogenous GLP-1 to activate the receptor, thereby improving glycemic outcomes and reducing body weight primarily by lowering energy intake through appetite suppression and delayed gastric emptying (Andreasen et al., 2021). Trials directly comparing GLP-1 RAs showed all agents produced reductions in hemoglobin A1c (HbA1c), ranging between 0.8% and 1.8%, with variable onset and magnitude of body weight reduction. Specifically in patients with T2DM, mean weight loss with GLP-1 RAs typically ranges from ∼2 to 7 kg across randomized controlled trials, with greater reductions (up to 15 kg with semaglutide) reported in obesity trials. While generally well tolerated, GLP-1 RAs are most frequently associated with gastrointestinal (GI) adverse events, including nausea, vomiting, and diarrhea, which represent a common cause of treatment discontinuation (Ismaiel et al., 2025).

Tirzepatide is a dual GIP and GLP-1 RA that gained U.S. Food and Drug Administration (FDA) approval for T2DM in adults and chronic weight management in adults with obesity (Tirzepatide, 2022).

Across the phase 3 SURPASS program, tirzepatide consistently demonstrated clinically relevant effects on glycemic control, with dose-dependent reductions in body weight in patients with T2DM. Overall, HbA1c reductions were in the range of 2.0–2.4 percentage points, accompanied by weight loss that frequently exceeded what has been reported with GLP-1 receptor agonists alone. In SURPASS-1, which evaluated tirzepatide as monotherapy compared with placebo in patients treated with diet and exercise, HbA1c decreased by nearly 2% and body weight was reduced by approximately 9 kg (Rosenstock et al., 2021).

Tirzepatide was compared directly with semaglutide 1 mg in the SURPASS-2 trial providing greater reduction in HbA1c (up to −2.30% vs. −1.86%) and larger decrease in body weight, with differences exceeding 5 kg, indicating superior efficacy to an established GLP-1 RA comparator (Frías et al., 2021).

When assessed against basal insulin regimens, tirzepatide demonstrated superiority in lowering HbA1c by more than 2 percentage points, while reducing body weight by up to 8–9 kg, in contrast to the weight gain typically observed with insulin degludec or insulin glargine, even in patients with elevated cardiovascular risk (Del et al., 2021; Dahl et al., 2022).Though efficacy outcomes favored treatment with tirzepatide, adverse events were more prominent in this cohort. Gastrointestinal effects, such as nausea and diarrhea, led to treatment discontinuation in approximately 2%–3% of tirzepatide recipients compared with lower rates in comparator groups. Episodes of hypoglycemia were also reported more often among patients receiving tirzepatide, particularly when used in combination with insulin.

Despite the increased risks of metabolic complications in solid organ transplant recipients (SOTRs), few studies have evaluated pharmacologic interventions in post-transplant patients, mainly because of the complexity of IS regimens and the potential drug–drug interactions. However, emerging evidence is considering the role of GLP-1 RAs and GLP-1/GIP RAs in this population given their broad metabolic benefits extending beyond glycemic control to include weight reduction and cardiovascular, renal, and hepatic protection. In general populations, GLP-1 RAs have consistently reduced HbA1c and body weight, and observational studies in transplant recipients, though limited by small sample sizes, have generally confirmed efficacy and safety without an excess of adverse events or pharmacological interactions with IS therapy. Tirzepatide, as the first dual GIP/GLP-1 receptor agonist, represents a promising therapeutic option in this context, with preliminary data suggesting clinically relevant improvements in metabolic outcomes. In this systematic review and meta-analysis, we therefore aim to assess the safety and efficacy of tirzepatide in SOTRs.

## 2 Methods

### 2.1 Study design and search strategy

The Preferred Reporting Items for Systematic Reviews and Meta-Analyses (PRISMA) 2020 guidelines were referenced when conducting this systematic review and meta-analysis (Page et al., 2021). A comprehensive search strategy, developed using the PICOS framework (Population, Intervention, Comparison, Outcome, and Study Design) (Higgins and Thomas, 2024).

This strategy was applied across the PubMed-MEDLINE, Cochrane Library, and Embase databases for relevant studies using a combination of database-specific subject headings and keywords. he following terms were used: (“tirzepatide” OR “dual GIP/GLP-1 receptor agonist” OR “GIP/GLP-1 RA”) AND (“transplant” OR “transplantation” OR “recipients” OR “SOT”). Only studies published from 1 May 2022, onward were considered, corresponding to the timing of FDA approval of tirzepatide (Eli Lilly and Company, 2022) The last literature search was conducted on 2 September 2025.

### 2.2 Inclusion and exclusion criteria

Studies that met the eligibility criteria were marked for inclusion:

- Population: Adults (≥18 years) who are SOT (kidney, liver, heart, lung, pancreas or combined), in the post-transplant period.
- Intervention: Treatment with tirzepatide (any dose), alone or with other antidiabetic agents, during the post-transplantation period.
- Outcome: Reported change in HbA1c and/or BMI/weight (efficacy); safety endpoints including treatment discontinuation due to adverse events.
- Study design: Randomized controlled trials (RCT) or observational studies (prospective/retrospective cohorts, registries).

Two authors (BM and RS) independently screened the studies that surfaced within the initial search and then identified studies for inclusion. Titles and abstracts were first screened for relevance, followed by a full-text screening using the eligibility criteria listed above to finalize studies to be included and analyzed. Any discrepancies were resolved by a third author (AP). Only full-text articles published in English from 1 May 2022 onward were eligible for inclusion.

To remove any duplicate studies, references were imported into the Rayyan® tool to be reviewed semi-automatically (Ouzzani et al., 2016). References were also manually reviewed for duplicates by two authors (BM and RS), confirmed, and removed.

Studies that focused on patients exclusively receiving another GLP-1 RA or SGLT2i or evaluated the role of tirzepatide in the pretransplant period were excluded. Any non-English articles without sufficient data were also excluded. To continue, systematic reviews and meta-analyses, clinical practice guidelines, case series, poster abstracts, animal studies, active clinical trials, and commentaries/editorials were excluded.

### 2.3 Data extraction

Data to be derived from included studies was determined by two reviewers (BM and RS), and the strategy was approved by a third author (AP), who oversaw the process. Three authors (BM, RS, and AP) independently extracted data on 16 September 2025 from each included study. The following data were extracted: (a) study author and year of publication; (b) study characteristics (including study design, time period, sample size, reasons for exclusion, prevention of bias methods, and funding); (c) patient characteristics (including median age, sex, race/ethnicity, comorbidities, type of SOT, time from transplant to tirzepatide initiation), median HbA1c at baseline, median body mass index (BMI) at baseline, use of other glycemic/diabetes medications, IS treatment at baseline, and tirzepatide treatment (including sample size, dosing, frequency, and duration); (d) types of outcome measurements; and (e) results for each outcome. For each included study, outcomes data were extracted at the latest available post–tirzepatide initiation assessment reported in the full text or provided by authors. When multiple follow-up windows were available, the longer window was preferentially selected for consistency. Due to heterogeneity in follow-up duration and reporting, no formal timepoint harmonization was performed. Adjustment for concomitant glucose-lowering therapies was not feasible due to aggregated reporting, lack of patient-level data, and the absence of comparator groups.

### 2.4 Outcomes

Primary outcomes of interest were defined as:

- Efficacy: changes in glycemic control, assessed by HbA1c and changes in weight, assessed by BMI.
- Safety: adverse drug reactions (ADRs), with particular focus on treatment discontinuation attributable to ADRs.

### 2.5 Synthesis method

Eligible studies were grouped for synthesis by systematically recording all reported outcomes. A summary table was created including the study identifiers, number of patients treated with tirzepatide, and efficacy and safety outcomes. Once tabulated, a visual comparison of results was performed, and the authors determined which outcomes were reported numerically in all four studies for inclusion in the synthesis.

### 2.6 Certainty assessment

The certainty of evidence was evaluated using the GRADE approach (Grading of Recommendation, Assessment, Development, and Evaluation) (Higgins and Thomas, 2024). Two authors (BM and RS) independently applied the GRADE methodology using the five GRADE domains of risk of bias, inconsistency, imprecision, indirectness, and publication bias.

Effective practice and organization of care (EPOC) (Cochrane, 2025) worksheets were utilized to aid in the creation of a summary of findings (SoF) table. Four levels of certainty could be assigned for each outcome, including high, moderate, low, or very low. Certainty of evidence was downgraded appropriately when necessary, and justifications were provided in the comments.

### 2.7 Statistical analysis

Meta-analysis of the difference of medians was conducted using the metamedian package in R (McGrath et al., 2024). The metamedian function implements the methods proposed by McGrath et al. (2019), McGrath et al. (2020) and Ozturk and Balakrishnan (2020) to estimate pooled differences in medians (McGrath et al., 2024). Specifically, it applies the (weighted) median of medians method. For two groups (baseline and follow-up), it implements the (weighted) median of the difference of medians method and the quantile matching estimation method.

Meta-analysis of adverse events was performed using the meta package in R (Balduzzi et al., 2019). The metaprop function was used to calculate the overall proportion from studies reporting a single proportion. A continuity correction of 0.5 was applied in studies with zero events. Results were reported as pooled proportions with 95% confidence intervals (95% CI). Heterogeneity was assessed through τ^2^, I^2^, and H statistics.

P-values less than 0.05 were considered statistically significant. All statistical analyses were conducted using R (R Core Team, 2024) (R Core Team, 2024).

Given the small number of studies, the absence of control groups, and the frequent reporting of medians without measures of dispersion, we adopted a weighted median of differences approach as a descriptive summary measure. This method was selected to reduce sensitivity to influential individual studies and to avoid assumptions required by mean-based pooling in extremely small and heterogeneous samples.

### 2.8 Risk of bias assessment

The qualitative risk of bias of included studies was assessed using the Risk of Bias in Non-randomized Studies of Interventions, Version 2 (ROBINS-I V2) tool (McGuinness and Higgins, 2020). The ROBINS-I V2 tool assesses the risk of bias for each study using 7 domains for follow-up studies that includes:

1. Confounding
2. Selection of participants into the study
3. Classification of interventions
4. Deviations from intended interventions
5. Missing data
6. Measurement of the outcome
7. Selection of the reported result

An overall risk-of-bias judgement for each individual bias domain can then be made and defined as low, moderate, serious, or critical risk of bias. Two independent reviewers (BM and RS) applied the ROBINS-I V2 tool to each individual study. Any discrepancies were then resolved by a third author (AP).

### 2.9 Publication bias

Publication bias was evaluated for the quantitative meta-analysis using funnel plots and radial plots and linear regression tests for funnel plot asymmetry:

- Egger’s regression test (Egger et al., 1997).
- Thompson and Sharp’s test (Thompson and Sharp, 1999).

Both statistical methods assess the presence of funnel plot asymmetry, which may indicate small-study effects or publication bias. Statistical significance was defined as *p* < 0.05.

Given the small number of included studies, publication bias analyses (funnel plots and regression-based tests) were conducted for exploratory purposes only, without inferential intent. Although these exploratory analyses are reported for transparency, current methodological guidance discourages interpretation of publication bias tests when very few studies are available; therefore, these results were not considered in the overall interpretation of the findings.

## 3 Results

### 3.1 Study selection

**FIGURE 1:**
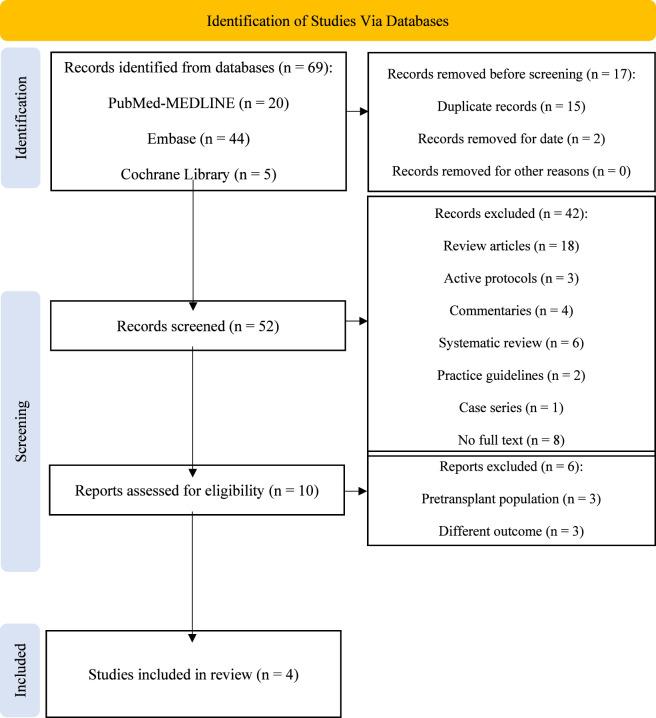
Preferred reporting items for systematic reviews and meta-analyses (PRISMA) Methodology Flowchart (Page et al., 2021).

The PRISMA flow diagram (Figure 1) outlines the study selection process. A total of 69 records were identified through PubMed, Embase, and the Cochrane Library. Before screening, 17 records were removed: 2 published prior to 1 May 2022 and 15 identified as duplicates using the Rayyan® detection tool. All removals were manually confirmed by cross-checking authors, titles, and publication dates. The remaining 52 records underwent screening by title and abstract, resulting in the exclusion of 42 records due to the wrong publication type, including 18 review articles, 3 active protocols, 4 commentaries, 6 systematic reviews, 2 practice guidelines, 1 case series, and 8 due to lack of full text availability. The 10 remaining records proceeded to full-text screening. Of these, 3 were removed due to different study populations and 3 due to different outcomes. Following thorough screening, 4 studies met the eligibility criteria and were included in the review for data extraction. Randomized controlled trials and other interventional clinical trials were not identified in the available literature and therefore were not included.

### 3.2 Baseline characteristics of the included studies

All were non-randomized, observational studies published between 2024 and 2025, specifically retrospective chart reviews. Three studies were conducted in the United States and one in the United Arab Emirates. Only two of the included studies focused exclusively on tirzepatide, whereas for the other two, supplemental tirzepatide-specific data were obtained directly from the authors and incorporated into our analysis.

Baseline characteristics of the four included studies (n = 86 patients) are summarized in Table 1 (Sweiss et al., 2025; Donald et al., 2024; January et al., 2025; El Khatib et al., 2025). Patients’ median age ranged from 58–59 years, with one study reporting a mean age of 60 years (Sweiss et al., 2025; Donald et al., 2024; January et al., 2025; El Khatib et al., 2025). Sex distribution was a total of 48 (55.8%) males and 37 (43%) females. One study was missing data for one participant regarding sex. Three studies included patients with pre-existing diabetes mellitus (DM). Most patients received a kidney or liver transplant, 44 and 21 patients, respectively; however, heart, lung, and combination transplant patients were also included in these studies. Time from transplant to tirzepatide initiation varied widely (range: 896 to 1,598 days). Baseline HbA1c ranged from 7% (53 mmol/mol) to 8.7% (72 mmol/mol), and baseline BMI ranged from 32.4 kg/m^2^ to 38.8 kg/m^2^. Some baseline data were missing, as detailed in Table 1. All but one study reported concomitant use of other glycemic medications, with most patients receiving insulin, metformin, and/or an SGLT2i. Only one study reported detailed immunosuppressive regimens; however, despite being included in the published article, immunosuppressant data specific to patients receiving tirzepatide were not available for all studies.

**TABLE 1:** Baseline characteristics of the included studies (Sweiss et al., 2025; Donald et al., 2024; January et al., 2025; El Khatib et al., 2025).

Study	Year	Design (country, study years)	No. of patients	Age (years as median), [*mean]	Sex, n (%)	Race, n (%)	Comorbidities, n (%)	Type of transplant, n (%)	Time from transplant to tirzepatide initiation (days as median)	BaselineHbA1c (% as median)	Baseline BMI (kg/m^2^ as median)	Use of other glycemic medications, n (%)	Maintenance immunosuppression
Sweiss et al.	2025	Retrospective chart review (United States, 2022–2023)	39	59	F: 17 (43.6) M: 22 (56.4)	Hispanic: 17 (43.6) Caucasian/White: 18 (46.1) African American: 1 (2.6) Other: 3 (7.7)	History previous transplant: 2 (5.1) History pre-transplant DM: 23 (58.9)	Kidney: 21 (53.8) Liver: 14 (35.9) Lung: 3 (7.7) Kidney-liver: 1 (2.6)	896	7**	32.7**	Insulin: 23 (58.9) Metformin: 9 (23.1) SGLT2i: 9 (23.1) Pioglitazone: 2 (5.1)	NR
Donald et al.	2024	Retrospective chart review (United States, 2010–2024)	5	58	F: 1 (20) M: 4 (80)	Hispanic: 1 (20) White 3: (60) Black 1: (20)	Hypertension: 4 (80) Hyperlipidemia: 5 (100) DM: 4 (80) CKD: 1 (20) Obesity: 5 (100)	Heart: 4 (80) Heart-kidney: 1 (20)	1,598	8.7	36.8	Insulin: 3 (60) SGLT2i: 2 (40)	NR
January et al.	2025	Retrospective chart review (United States, 2018–2025)	8	60*	F: 3** (42.9) M: 4** (57.1)	NR	NR	Lung: 8 (100)	1,071	7.05**	38.8	NR	NR
El Khatib et al.	2025	Retrospective chart review (United Arab Emirates, 2017–2024)	34	58.5	F: 16 (47.1) M: 18 (52.9)	NR	History Type 2 DM: 31 (91.2) CKD stage 1: 6 (17.6) CKD stage 2: 18 (52.9) CKD stage 3: 8 (26.5) CKD stage 4: 1 (2.9)	Heart: 2 (5.9) Kidney: 23 (67.6) Liver: 7 (20.6) Lung-kidney: 1 (2.9) Simultaneous pancreas-kidney: 1 (2.9)	1,062	8.3	32.44	Metformin: 18 (47.4) SGLT2i: 16 (45.6) Sulfonylurea: 5 (14.3) Meglitinide: 2 (5.7)	Tacrolimus: 32 (94.1) Cyclosporine: 2 (5.9) Everolimus: 2 (5.9) Prednisolone: 23 (67.6) Mycophenolate mofetil: 29 (85.3)

NR, not reported; F, female; M, male; DM, diabetes mellitus; CKD, chronic kidney disease; BMI, body mass index; HbA1c, hemoglobin A1c; SGLT2i, sodium-glucose cotransport 2 inhibitor.

*Value reported as mean, **baseline data reported with missing participants.

### 3.3 Results of individual studies

Tabulated results for efficacy and safety outcomes are represented in Table 2.

**TABLE 2:** Studies on tirzepatide in post-transplant patients: association of tirzepatide and safety and efficacy outcomes (Sweiss et al., 2025; Donald et al., 2024; January et al., 2025; El Khatib et al., 2025).

Study ID	Change in BMI (kg/m^2^ as median)	Change in body weight (kg as median)	Change in HbA1c (% as median)	Change in FBG (mg/dL as median)	Change in SCr (mg/dL as median)	Change in LDL (mg/dL as median)	Change to eGFR (mL/min/1.732 as median)	Change in TG (mg/dL as median)	Change to NT-proBNP (pg/mL as median)	Change in insulin requirements (units as median)	Discontinuation due to adverse drug reactions (rationale)	Adverse event type (no. of events)
Sweiss et al., 2025 (n = 39)* (Sweiss et al., 2025)	↓ 1.7 (n = 20)	↓ 3.4 (n = 20)	↓ 0.35 (n = 9)	↓ 23 (n = 18)	↓ 0.04 (n = 21)	NR	NR	NR	NR	NR	1 (severe GI intolerance)	Nausea/vomiting (2) hypoglycemia (2)
Donald et al., 2024 (n = 5) (Donald et al., 2024)	↓ 1.1	NR	↓ 1.7	NR	↑ 0.1**	↓ 34**	↓ 6**	↓ 77**	↓ 43	↓ 8**	0	NR
January et al., 2025 (n = 8) (January et al., 2025)	↓ 5.9	↓ 10	↓ 1.2 (n = 6)	NR	NR	NR	NR	NR	NR	NR	0	GI issues (3), headache (2), fatigue (4)
El Khatib et al., 2025 (n = 34) (El Khatib et al., 2025)	↓ 1.15	↓ 5.5	↓ 1.4	↓ 3.9	↓ 4	NR	0	↓ 0.96	NR	↓ 12	2 (severe GI intolerance)	Ketone bodies in urine (10), severe GI intolerance resulting in discontinuation (2), ED visits due to GI intolerance or hypoglycemia (8), all cause hospital admission (6), infection (3)

*data from 1-to-12-month Nadir; **missing follow-up data, does not include all participants; HbA1c, hemoglobin A1c; BMI, body mass index; NR, not reported; FBG, fasting blood glucose; SCr, serum creatinine; LDL, low density lipoprotein; TG, triglyceride; eGFR, estimated glomerular filtration rate; NT-proBNP, N-terminal pro b-type natriuretic peptide; GI, gastrointestinal; ED, emergency department.

Sweiss et al. (2025) conducted a single-center retrospective chart review of 39 adult transplant recipients prescribed tirzepatide for preexisting T2DM or PTDM. The electronic medical record was screened for recipients of a kidney, liver, and lung, excluding those with a history of pancreas transplant, type 1 diabetes, nonadherence to tirzepatide, or insufficient follow-up. Efficacy of tirzepatide was assessed using fasting blood glucose (FBG), HbA1c, body weight, BMI, and serum creatinine (SCr). “Nadirs” were defined in the original study as the lowest laboratory values obtained from patient charts within the 1–6 months and 1–12 months periods and were compared with baseline values. Safety was assessed over 6 months by graft failure, hospitalizations, and reporting of adverse events, including nausea/vomiting, pancreatitis, and hypoglycemia. At the 1- to 6-month nadir, statistically significant reductions were observed across all outcomes. At the 1- to 12-month nadir, FBG decreased from 132 mg/dL to 109 mg/dL, body weight decreased from 97.5 kg to 94.1 kg, and BMI decreased from 32.7 kg/m^2^ to 31.0 kg/m^2^. These reductions remained statistically significant, while changes in HbA1c (7.0% [53 mmol/mol] to 6.65% [49 mmol/mol], p = 0.2266) and SCr (1.06 mg/dL to 1.02 mg/dL, p = 0.0784) did not reach significance. Tirzepatide was discontinued in two patients due to insurance issues, and one patient discontinued due to gastrointestinal (GI) adverse effects. During the 6-month safety follow-up, no cases of pancreatitis, graft failure, or hypoglycemia requiring hospitalization were reported, and serum creatinine remained stable, suggesting preserved graft function.

Donald and colleagues (Donald et al., 2024) retrospectively reviewed the electronic medical records of adult heart transplant recipients who were prescribed a GLP-1 RA or GLP-1/GIP RA post-transplant. Patients were excluded if therapy lasted less than 1 month, if the medication was never initiated, or if it was not resumed post-transplant. While the authors included semaglutide, liraglutide, dulaglutide, and tirzepatide in their published analysis, the authors were contacted and provided supplemental data for only the 5 patients on tirzepatide treatment. Changes to HbA1c and BMI were included as primary outcomes of interest, as well as changes to low-density lipoprotein (LDL) and triglycerides (TG). Medians and interquartile ranges were reported for non-normally distributed data, with p < 0.05 considered statistically significant. Among tirzepatide recipients, median HbA1c decreased by 1.7% (from 8.7% [72 mmol/mol] to 7.0% [53 mmol/mol]) and median BMI decreased by 1.1 kg/m^2^ (36.8 kg/m^2^ to 35.7 kg/m^2^) from baseline. There were also decreases to median LDL and TG values from baseline; however, only data for 3 patients post-tirzepatide treatment was provided. Secondary outcomes also included changes to estimated glomerular filtration rate (eGFR), N-terminal pro b-type natriuretic peptide (NT-proBNP), SCr, and changes to insulin requirements. As reflected in Table 2, median values of eGFR, NT-proBNP, and insulin requirements decreased; however, there was an increase in median SCr. The follow-up data for these secondary outcomes also was missing data for all participants. The author reported that no patients on tirzepatide discontinued treatment due to adverse events. Regarding safety, no tirzepatide-treated patients discontinued therapy due to adverse events, and no cases of pancreatitis, severe hypoglycemia, or hospitalizations related to treatment were reported. Renal function and cardiac biomarkers remained largely stable during follow-up, and no clinically relevant changes in calcineurin inhibitor exposure were attributed to tirzepatide.

In January et al. (2025) a retrospective review was conducted for 81 adult lung transplant recipients who received a GLP-1 RA or GLP-1/GIP RA for weight loss, diabetes, or both. Patients without spirometry testing after therapy or those who began the agent fewer than 90 days post-transplant were excluded. In the published cohort, patients were treated with dulaglutide, semaglutide, liraglutide, and tirzepatide, but the author provided supplemental materials for the 8 tirzepatide recipients. The primary objective was to determine whether weight loss was associated with improved lung function, measured by changes in forced expiratory volume (FEV_1_) and forced vital capacity (FVC) from initiation to completion of GLP-1 based therapy. Secondary objectives included weight change, HbA1c reduction, and incidence of adverse events. In the full cohort of 81 patients, increases were observed in nearly half of patients for FEV1 (49%) and FVC (46%), but results did not reach statistical significance. A subgroup analysis was conducted for patients with a BMI ≥30 kg/m^2^, and a sensitivity analysis excluded patients who experienced any degree of rejection. Both analyses, however, were limited to the full cohort. Among the 8 patients treated with tirzepatide, all achieved clinically meaningful weight loss. Median HbA1c improved from 7.05% (53 mmol/mol) at baseline to 5.85% (41 mmol/mol) at the conclusion of the study. Median BMI decreased from 38.8 kg/m^2^ at baseline to 32.9 kg/m^2^ at day 300 of therapy. Reported adverse events included gastrointestinal effects, headache, and fatigue, but none warranted discontinuation. No cases of acute kidney injury, pancreatitis, severe hypoglycemia, or graft rejection were reported among tirzepatide-treated patients. Data on emergency department visits, hospitalizations, and calcineurin inhibitor trough levels were not systematically reported in this study.

El Khatib et al. (2025) conducted a retrospective medical record review of patients who received tirzepatide treatment post-transplant, excluding those who received treatment for less than 3 months post-transplant. To evaluate efficacy, changes in body weight, BMI, TG, insulin requirements, HbA1c, FBG, and eGFR were calculated. Nonparametric values were reported as median and interquartile ranges while categorical variables were presented as frequencies and percentages. A p-value <0.05 was considered statistically significant. For dosing, tirzepatide was initiated on 2.5 mg once weekly and titrated in 2.5-mg increments, with a median dose of 5 mg at study end. Only 1 patient reached the maximum weekly dose of 15 mg during the study period. The median treatment duration was 11 months. Among the 34 patients included (2 heart, 23 kidney, 7 liver, 1 lung-kidney, 1 simultaneous pancreas-kidney), tirzepatide significantly reduced median body weight by 6.5% (85 kg–79.5 kg, p < 0.001) and median BMI by 1.15 kg/m^2^ (32.4 kg/m^2^ to 31.3 kg/m^2^, p < 0.001). Reductions to median HbA1c and FBG compared to baseline also demonstrated statistical significance through decreases of 1.4% (8.3% [67 mmol/mol] to 6.9% [52 mmol/mol]) and 3.9 mmol/L (10.55 mmol/L to 6.65 mmol/L), respectively (p < 0.001). Total daily insulin requirements decreased from 46 units to 34 units (p = 0.01). TG levels also decreased significantly from baseline with tirzepatide (2.48 mg/dL to 1.52 mg/dL) whereas SCr was unchanged. eGFR significantly improved (p = 0.04). The authors also performed a subgroup analysis to determine the effect of tirzepatide on kidney transplant recipients compared to other types of SOTs. They found that reductions to BMI, HbA1c, and FBG, and improvements to eGFR, were not significantly different between the type of SOT; however, tirzepatide resulted in significant increases in SCr in non-kidney transplant patients. For safety, 23.5% (n = 8) of patients required visits to the emergency department (ED) due to GI intolerance or hypoglycemia, which led to hospital admission for 17.6% (n = 6) of these patients. Only 2 patients discontinued tirzepatide therapy due to severe GI intolerance. No cases of pancreatitis, graft rejection, or patient mortality were reported. Importantly, no clinically meaningful changes in steady-state tacrolimus trough levels were observed after tirzepatide initiation, despite the known effects of GLP-1/GIP receptor agonists on gastric emptying.

### 3.4 Statistical analysis

#### 3.4.1 HbA1c reduction

**FIGURE 2:**
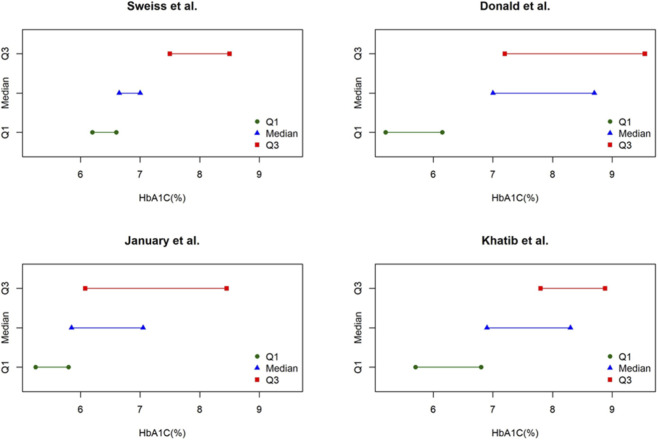
Study-level distribution of Hb1Ac values at baseline and follow-up (medians and interquartile ranges). Each panel corresponds to one study. Symbols represent the quartiles: green circles = Q1 (25th percentile), blue triangles = median (50th percentile), red squares = Q3 (75th percentile). Horizontal lines indicate the spread of values within each quartile. Data illustrate the distribution of HbA1c at baseline and follow-up for each included study.

Four studies (*n* = 5–34 per group) contributed to the analysis of HbA1c. Median baseline values ranged from 7.0% (53 mmol/mol) to 8.7% (72 mmol/mol), while follow-up medians ranged from 5.85% (41 mmol/mol) to 7.0% (53 mmol/mol). Using the Weighted Median of the Difference of Medians method, the pooled estimate indicated a significant reduction of −1.4% (95% CI: −1.7 to −0.4). This decrease is clinically meaningful, as a reduction of around 1% in HbA1c is generally associated with a lower risk of diabetes-related complications (Authors, 1999). Consistent declines across the included studies support the robustness of this finding (Table 3; Figure 2).

**TABLE 3:** HbA1c at baseline and follow-up, with median differences.

Study	N patients	Baseline HbA1c % (median, 25th–75th percentile)	Follow-up HbA1c % (median, 25th–75th percentile)	Δ Median (%)
Sweiss et al.	9	7.0 (6.6–8.5)	6.65 (6.2–7.5)	−0.35
Donald et al.	5	8.7 (6.15–9.55)	7.0 (5.2–7.2)	−1.70
January et al.	6	7.05 (5.8–8.45)	5.85 (5.25–6.08)	−1.20
Khatib et al.	34	8.3 (6.8–8.88)	6.9 (5.7–7.8)	−1.40
Pooled estimate	—	—	—	−1.4 (95% CI: −1.7 to −0.4)

Values are medians (25th–75th percentile). Δ Median represents the change from baseline to follow-up. The pooled difference was estimated using median-based meta-analysis. Negative values indicate an improvement in glycemic control. All studies consistently showed reductions in HbA1c values.

#### 3.4.2 BMI reduction

**FIGURE 3:**
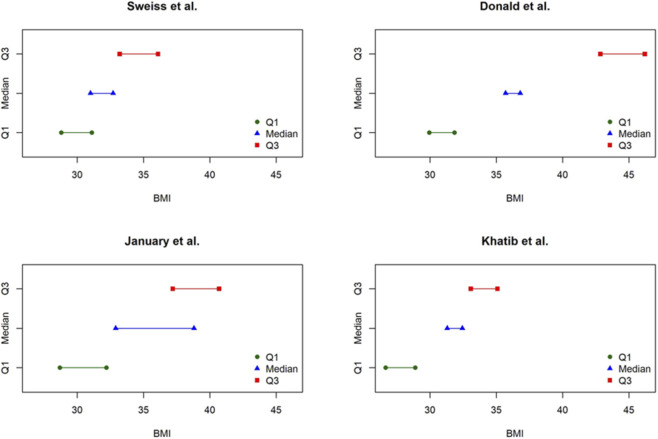
Study-level distribution of BMI values at baseline and follow-up (medians and interquartile ranges). Each panel corresponds to one study. Symbols represent the quartiles: green circles = Q1 (25th percentile), blue triangles = median (50th percentile), red squares = Q3 (75th percentile). Horizontal lines indicate the spread of values within each quartile. Data illustrate the distribution of BMI at baseline and follow-up for each included study.

Four studies (*n* = 5–34 per group) contributed to the analysis of BMI. Median BMI values at baseline ranged from 32.4 to 38.8 kg/m^2^, while follow-up values ranged from 31.0 to 35.7 kg/m^2^. Using the Weighted Median of the Difference of Medians method, the pooled estimate indicated a significant reduction of −1.2 units (95% CI: −5.9 to −1.1). Given the small number of included studies, formal influence diagnostics were not planned a priori. Robustness was therefore assessed descriptively by examining the contribution of individual studies to the pooled estimate. The wide confidence interval reflects substantial between-study heterogeneity, with the largest influence driven by the lung-transplant cohort reported by January et al. Nevertheless, BMI decreased from baseline in all included studies, suggesting a consistent directional signal toward modest BMI reduction across solid organ transplant recipients treated with tirzepatide (Table 4; Figure 3).

**TABLE 4:** BMI at baseline and follow-up, with median differences.

Study	N. of patients	Baseline BMI (25th–75th percentile)	Follow-up BMI (25th–75th percentile)	Δ Median
Sweiss et al.	20	32.7 (31.1–36.1)	31.0 (28.8–33.2)	−1.7
Donald et al.	5	36.8 (31.85–46.2)	35.7 (29.95–42.85)	−1.1
January et al.	8	38.8 (32.2–40.7)	32.9 (28.7–37.2)	−5.9
Khatib et al.	34	32.44 (28.89–35.09)	31.29 (26.65–33.08)	−1.15
Pooled estimate	—	—	—	−1.2 (95% CI: −5.9 to −1.1)

Values are medians (25th–75th percentile). Δ Median represents the change from baseline to follow-up. The pooled difference was estimated using median-based meta-analysis. Negative values indicate a reduction in BMI.

#### 3.4.3 Adverse drug reactions

**FIGURE 4:**
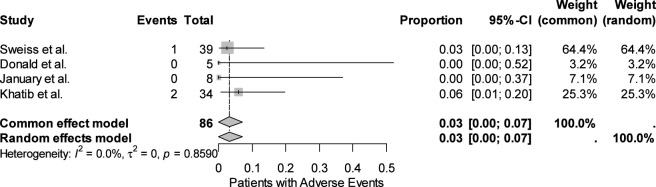
Forest plot: Patients who discontinued tirzepatide treatment due to ADRs.

**FIGURE 5:**
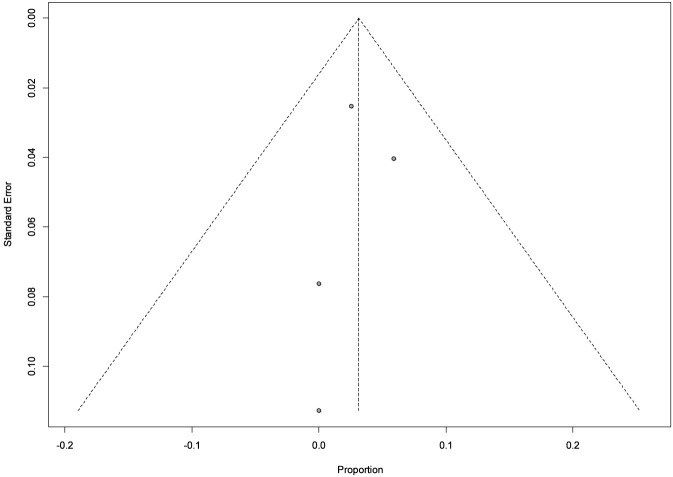
Funnel plot: Patients who discontinued tirzepatide treatment due to ADRs.

**FIGURE 6:**
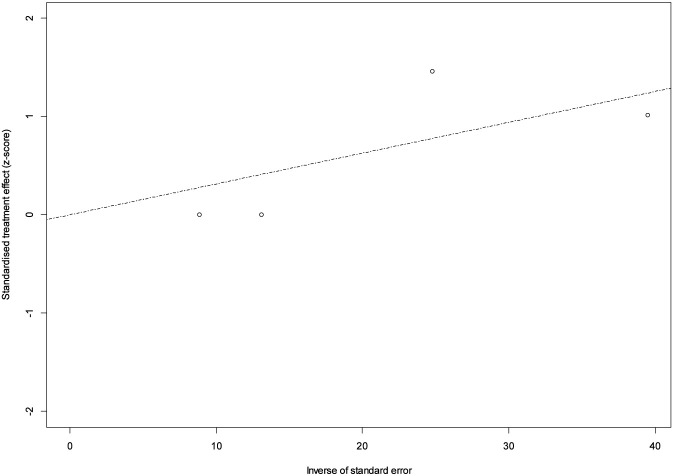
Radial plot: Patients who discontinued tirzepatide treatment due to ADRs.

Safety data were reported by all four studies, encompassing 86 participants. Only 3 adverse events leading to treatment discontinuation with tirzepatide were recorded overall, corresponding to a pooled event rate of 3.1% (95% CI: 0.0–7.1). Both common-effect and random-effects models yielded identical results, with no evidence of heterogeneity (I^2^ = 0.0%; p = 0.859) (Figure 4). Funnel plot asymmetry analyses did not indicate publication bias (Egger test: p = 0.72; Thompson & Sharp test: p = 0.83) (Figure 5). Although the absence of bias signals supports the robustness of these findings, the small number of studies limits the sensitivity of such tests. Radial plot inspection confirmed the absence of outlier studies contributing to heterogeneity. This suggests that the interventions under study were well tolerated, with a low and consistent risk of adverse outcomes across populations (Figure 6).

However, the absence of publication bias signals supports the robustness of the findings, although the small number of studies limits the sensitivity of such tests.

This meta-analysis, using methods tailored for median-based data, found significant reductions in both BMI and HbA1c at follow-up compared with baseline, with HbA1c improvements being clinically meaningful. Adverse events were rare and showed no heterogeneity across studies, supporting a favorable safety profile. While the results are promising, the limited number of small studies warrants cautious interpretation and highlights the need for larger, patient-level investigations to confirm these findings.

### 3.5 Risk of bias assessment

**FIGURE 7:**
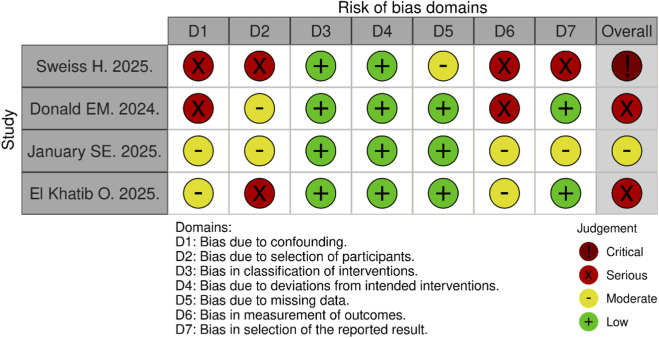
Overall risk of bias assessment stratified by study using the ROBINS-I tool, chart was created using Risk-of-bias VISualization (ROBVIS) (McGuinness and Higgins, 2020).

**FIGURE 8:**
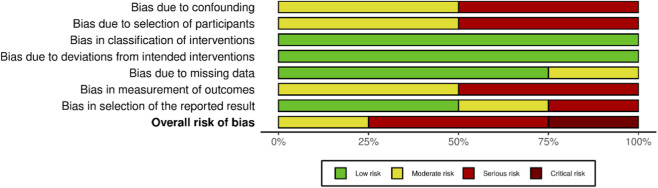
Overall risk of bias assessment stratified by domain using the ROBINS-I tool, chart was created using Risk-of-bias VISualization (ROBVIS) (McGuinness and Higgins, 2020).

Four non-randomized studies evaluating the safety and efficacy of tirzepatide were included in the risk of bias assessment. Using the ROBINS-I V2 tool, one study was judged to have a moderate risk of bias, two as having a serious risk of bias, primarily due to unmeasured confounding and participant selection, and one as having a critical risk of bias (Figure 7). The critical-risk study had four domains assessed as serious due to significant confounding, participant selection, and selective reporting due to convenience of follow-up. Overall, 75% of the studies were found to have a serious or critical risk of bias, while only one study was judged as moderate (Figure 8).

**FIGURE 9:**
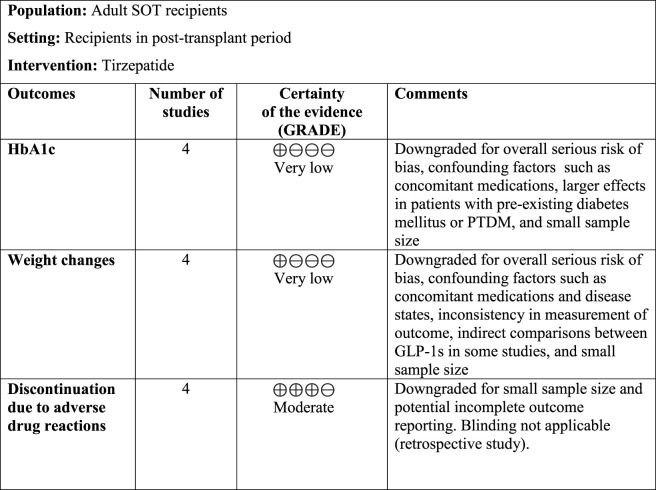
SoF of the certainty assessment with GRADE. The evidence is classified into high, moderate, low, and very low certainty.

The Summary of Findings (SoF) (Figure 9) presents the certainty of evidence based on GRADE criteria for outcomes including HbA1c, weight change, and discontinuation due to adverse events. Across all outcomes, the certainty of evidence was downgraded due to serious risk of bias, small sample size, and the lack of control groups. HbA1c and weight changes were classified as having very low certainty due to the influence of concomitant medications such as SGLT2i and insulin and differences related to underlying conditions such as obesity, diabetes mellitus, or both. Discontinuation due to adverse events was judged to have moderate certainty due to standardized reporting.

## 4 Discussion

To our knowledge, this is the first systematic review and meta-analysis to specifically evaluate the safety and efficacy of tirzepatide in solid organ transplant recipients. The evidence included in the current study suggests that tirzepatide reduces HbA1c and BMI in SOTRs without causing a significant number of ADRs that lead to discontinuation of therapy.

All 4 included studies suggest that tirzepatide contributed to reductions in HbA1c and BMI in different types of transplants, including heart, liver, kidney, lung, and pancreas recipients (Tables 1, 2). Demonstrated reductions in HbA1c are clinically relevant for the management of PTDM, a prevalent post-transplant complication. While therapeutic recommendations for PTDM largely mirror those for T2DM, tirzepatide shows minimal potential for drug–drug interactions with immunosuppressants and does not require dose adjustments in renal or hepatic impairment, supporting its role as a suitable therapeutic option in this population. Beyond immunosuppressive therapy, lifestyle changes after transplantation, including increased appetite and relaxation of dietary restrictions, frequently contribute to weight gain and accumulation of visceral adiposity, with subsequent metabolic complications. The consistent effect on BMI supports the potential use of tirzepatide in managing obesity and related post-transplant metabolic disorders associated with PTDM, with expected benefits not only for quality of life and graft function but also for cardiovascular risk reduction through improved metabolic control.

Furthermore, tirzepatide was generally well tolerated, with only a small number of participants discontinuing treatment due to severe GI intolerance. Our findings are consistent with those of the general population, where discontinuations are primarily related to nausea and diarrhea (Rosenstock et al., 2021).

Tirzepatide has been extensively investigated in the general population with T2DM. A meta-analysis of the SURPASS program confirmed HbA1c reductions of 2.0–2.4 percentage points and body weight loss often exceeding 10 kg, with consistent benefits across different patient groups (Rosenstock et al., 2021). Furthermore, pooled analyses suggested a neutral to favorable effect on major adverse cardiovascular events (MACE), which is now being prospectively evaluated in the SURPASS-CVOT trial comparing tirzepatide with dulaglutide (Nicholls et al., 2024). These important benefits are potentially translatable to the population of SOTRs; however, as highlighted in the ADA Standards of Care, large and dedicated randomized controlled trials are required to confirm their efficacy and safety in this setting.

Beyond our analysis, large observational studies and systematic reviews have recently explored the role of GLP-1 receptor agonists (GLP-1 RAs) in SOTRs (Lin et al., 2025). A nationwide cohort study including 18,016 kidney transplant recipients with diabetes (of whom 1,969 received GLP-1 RAs after transplantation) reported that GLP-1 RA therapy was associated with a significantly lower risk of death-censored graft loss and all-cause mortality, without evidence of harm to graft function (Orandi et al., 2025).

Similarly, a 2025 systematic review and meta-analysis including more than 7,800 kidney transplant recipients receiving SGLT2 inhibitors or GLP-1 RAs found significant improvements in HbA1c and body weight, with no detrimental effects on renal function, further supporting the feasibility and safety of incretin-based therapy in this population (Lee et al., 2025).

Although neither study directly evaluated the dual GIP/GLP-1 receptor agonist tirzepatide, the latter confirmed the established benefits of SGLT2 inhibitors—such as robust cardiovascular and renal protection, reduced mortality, and improved graft outcomes—providing a rationale for further investigation of novel incretin-based therapies in transplant recipients.

These findings contribute to one of the potential biases that may limit the results of our meta-analysis. Indeed, among the studies we analyzed, the most important confounding variable was patients’ use of other glycemic medications for T2DM or PTDM. While this variable was not reported for one study, most patients in the other three studies were taking insulin, metformin, or SGLT2is in addition to tirzepatide. The studies with reported data for the use of these medications failed to adjust for this confounder, and reductions to HbA1c attributed to tirzepatide might be an overestimation. However, given their complementary mechanisms, co-administration of tirzepatide and SGLT2 inhibitors may provide synergistic benefits in the management of PTDM, obesity, and associated cardiovascular and renal complications in SOTRs.

In addition, these glycemic medications, especially metformin, could have contributed to the GI intolerance that caused patients to discontinue therapy (American Diabetes Association Professional Practice Committee. 9, 2025). While publication bias was absent and supports the tolerability of tirzepatide, the small number of studies limits the sensitivity of such tests.

Our literature search did not identify any full-text systematic reviews or meta-analyses evaluating the role of tirzepatide in SOTRs. We did, however, identify one prior systematic review available only as a poster abstract assessing GLP-1 RAs, including tirzepatide, in liver transplant recipients with diabetes (Trieu et al., 2025). Trieu and colleagues utilized 7 poster abstracts and 8 retrospective or pretest-posttest studies to determine the impact of several GLP-based therapeutics on glycemic control, weight loss, and cardiovascular outcomes. Their review is not directly comparable with ours, as we focused solely on tirzepatide, a dual GLP-1/GIP RA with a distinct mechanism of action that may offer additive benefits compared to GLP-1 RA alone. Despite an increased sample size, pooling outcomes across GLP-1 RA agents introduce heterogeneity and limits clinical applicability. We also extended our analyses beyond efficacy outcomes to assess safety and tolerability, which are particularly important considerations for SOTRs with complex IS regimens. Several relevant retrospective studies were available only as poster abstracts with no corresponding full text at the time of our review. Inclusion of these studies may have increased our sample size and strengthened our certainty of evidence.

## 5 Limitations

Despite these positive results, there were several limitations to our study. All studies included in our analysis were observational, contributing to a high likelihood of risk of bias. Any assessment of publication bias should be interpreted with extreme caution, as such analyses are severely underpowered with fewer than 10 studies and were performed for exploratory purposes only. Also, retrospective study designs introduce a serious limitation, as study investigators are aware of the intervention and study participants’ demographics before and during the study. The included chart reviews only reported data for a small number of patients, limiting our ability to draw significant conclusions about tirzepatide specifically in the post-transplant population. Two included studies also analyzed other GLP-1 RAs, demonstrating that tirzepatide is not the most prescribed medication in SOTRs and further limiting the achievable sample size. In addition, the heterogeneity in follow-up duration across studies represents an important limitation. Pooling baseline–follow-up contrasts at different timepoints may introduce bias and limits the interpretation of pooled estimates as time-specific effects. Therefore, results should be interpreted as indicative of short-to mid-term metabolic changes rather than definitive estimates of treatment efficacy over a standardized follow-up.

Participants with longer follow-up may therefore have demonstrated greater changes in BMI and HbA1c than those with shorter exposure durations, and no sensitivity analyses were performed to adjust for these differences.

There was also significant heterogeneity in reported outcomes. While efficacy and safety measures were generally consistent, other renal and metabolic parameters were not uniformly assessed or were reported using different laboratory metrics. The wide confidence interval observed for BMI change was largely driven by the lung-transplant cohort reported by January et al. (n = 8), which demonstrated a markedly larger reduction compared with other studies. This disproportionate influence reflects both clinical and methodological heterogeneity and limits the precision of the pooled BMI estimate.

The use of a weighted median of differences for pooling represents a pragmatic but limited approach in the setting of very small and heterogeneous observational studies. While this method reduces sensitivity to extreme values, it does not overcome the fundamental limitations related to study design, confounding, and lack of standardized follow-up, and therefore the clinical interpretability of pooled estimates remains limited.

Overall, tirzepatide was associated with consistent signals of HbA1c reduction and weight loss in solid organ transplant recipients; however, these findings should be interpreted with caution given substantial heterogeneity, potential confounding from concomitant treatment with other glucose-lowering agents represents a major source of confounding. Without adjustment or comparator groups, the observed metabolic changes may be partially or entirely driven by background therapies, leading to potential overestimation of the apparent effect of tirzepatide.

Finally, a major limitation of the current literature is the lack of detailed immunosuppressive regimen data across most included studies. Given the potential for pharmacokinetic interactions and the central role of immunosuppression in transplant outcomes, this limits the ability to fully assess drug–drug interactions, safety, and residual confounding in solid organ transplant recipients treated with tirzepatide.

The lack of control groups across all included studies represents a major limitation. Pre–post comparisons without comparators cannot distinguish treatment-related effects from regression to the mean or from the natural progression of metabolic outcomes, substantially limiting causal interpretation. The very low certainty of evidence according to GRADE reflects serious limitations related to study design, small sample size, heterogeneity, and confounding. Accordingly, conclusions regarding metabolic benefits should be interpreted with caution and should not be considered sufficient to support clinical applicability.

However, taken together, these findings suggest that tirzepatide may represent a promising therapeutic option for obesity and PTDM in solid organ transplant recipients, irrespective of transplant type; however, confirmation from larger, prospective, and adequately controlled studies is required before definitive conclusions can be drawn.

## 6 Conclusion

In conclusion, tirzepatide was associated with reductions in HbA1c and body weight in SOTRs, with generally tolerable adverse events. These findings suggest potential utility of tirzepatide forthe management of obesity and PTDM in this population; however, given the small sample sizes, retrospective design, heterogeneity of transplant types, and potential confounding from concomitant medications, these results should be interpreted with caution.

Tirzepatide appears to be safe with respect to renal function in the included studies, including in patients with advanced CKD or ESRD, but larger and prospective studies are required to confirm these observations. Randomized controlled trials comparing tirzepatide to other GLP-1 receptor agonists in the management of PTDM and obesity among transplant recipients would provide more robust evidence to guide clinical practice.

## Data Availability

The original contributions presented in the study are included in the article/supplementary material, further inquiries can be directed to the corresponding author.
