# Correction: Safety and efficacy of tirzepatide in transplant recipients: a systematic review and meta-analysis

**DOI:** 10.3389/fphar.2026.1834664

**Published:** 2026-04-29

**Authors:** Alessio Provenzani, Brinn Mancuso, Riley Stitch, Fabio Tuzzolino, Maria Ausilia Giusti, Alessandro Mattina

**Affiliations:** 1 IRCCS ISMETT, Palermo, Italy; 2 UPMC Italy, Palermo, Italy; 3 Department of Pharmacy and Therapeutics, University of Pittsburgh School of Pharmacy, Pittsburgh, PA, United States

**Keywords:** adverse drug reactions, efficacy, safety, tirzepatide, transplant recipients

In the published article, the citations for references Lin et al. (2025), Orandi et al. (2025), Lee et al. (2025) and Trieu et al. (2025) were misplaced. A correction has been made to section 4 **Discussion**, paragraphs 5 and 6:

“Beyond our analysis, large observational studies and systematic reviews have recently explored the role of GLP-1 receptor agonists (GLP-1 RAs) in SOTRs (Lin et al., 2025). A nationwide cohort study including 18,016 kidney transplant recipients with diabetes (of whom 1,969 received GLP-1 RAs after transplantation) reported that GLP-1 RA therapy was associated with a significantly lower risk of death-censored graft loss and all-cause mortality, without evidence of harm to graft function (Orandi et al., 2025).

Similarly, a 2025 systematic review and meta-analysis including more than 7,800 kidney transplant recipients receiving SGLT2 inhibitors or GLP-1 RAs found significant improvements in HbA1c and body weight, with no detrimental effects on renal function, further supporting the feasibility and safety of incretin-based therapy in this population (Lee et al., 2025).”

A correction has also been made to section **4 Discussion**, paragraph 10:

“We did, however, identify one prior systematic review available only as a poster abstract assessing GLP-1 RAs, including tirzepatide, in liver transplant recipients with diabetes (Trieu et al., 2025). Trieu and colleagues utilized 7 poster abstracts and 8 retrospective or pretest-posttest studies to determine the impact of several GLP-based therapeutics on glycemic control, weight loss, and cardiovascular outcomes.”

The original article has been updated.

